# Relationship between myeloid skewing and colorectal cancer risk in the Framingham Heart Study

**DOI:** 10.3389/fonc.2026.1773537

**Published:** 2026-04-22

**Authors:** Lisa Ek Orloff, Michael De Lisio

**Affiliations:** 1 School of Human Kinetics, University of Ottawa, Ottawa, ON, Canada; 2 Department of Cellular and Molecular Medicine, University of Ottawa, Ottawa, ON, Canada; 3 Regenerative Medicine Program, Éric Poulin Centre for Neuromuscular Disease, University of Ottawa, Ottawa, ON, Canada

**Keywords:** colorectal cancer, Framingham Heart Study, myeloid skewing, obesity, physical activity

## Abstract

**Introduction:**

Differential regulation of myelopoiesis may contribute to divergent colorectal cancer (CRC) risk profiles associated with obesity and physical activity; however, this relationship has not been examined in humans. This study explored the relationship between myeloid skewing (monocyte-to-lymphocyte ratio), physical activity, obesity, and CRC risk in a retrospective cohort.

**Methods:**

From the Framingham study cohort, myeloid skewing was compared across BMI categories, waist circumference quartiles, and physical activity index (PAI) quintiles. Among participants who eventually received a CRC diagnosis, Cox proportional hazards models evaluated baseline circulating myeloid skewing as a predictor of incident CRC, adjusted for covariates.

**Results:**

Within the total study population (n = 5037), myeloid skewing increased across BMI categories, and waist circumference quartiles (P <.001), and decreased in the highest vs lowest PAI (P <.01). In the predictive cohort (non-CRC, n = 818; CRC, n = 16), continuous Cox models (per 1-SD increase in myeloid skewing) showed hazard ratios (HR) ranging from 1.08 to 1.37. Associations were statistically significant in BMI, waist circumference, BMI and waist circumference, PAI, diabetes, sex, and smoking adjusted models (HRs 1.30-1.37; P <.05), but were attenuated and not statistically significant in age, age and sex, and medication adjusted models (HRs 1.08-1.28; P >.05).

**Discussion:**

Obesity and lower physical activity were associated with higher myeloid skewing, and higher myeloid skewing was associated with increased incident CRC hazard across several adjusted Cox models. These findings highlight myeloid skewing as a candidate marker linking adiposity and physical inactivity to CRC risk and justify prospective validation in larger cohorts.

## 1 Introduction

Colorectal cancer (CRC) is the third most commonly diagnosed cancer type in Canada, the second leading cause of cancer deaths (1), and is increasing among young adults who are below the age for regular preventative screening (2). This increasing CRC prevalence is suggested to be at least partly related to the rise in obesity and physical inactivity (2, 3). Conversely, participating in physical activity, including regular exercise, is associated with decreased CRC risk, reduced colorectal inflammation, and reduced mortality in patients with CRC (3, 4). Chronic inflammation is both a hallmark and a risk factor of cancer (5), and is differentially regulated by obesity and physical activity (6), suggesting that inflammatory pathways may be responsible for the associations of obesity and physical activity with CRC risk.

Hematopoietic stem and progenitor cell (HSPC) differentiation contributes crucially to peripheral inflammatory response by producing a balance of myeloid and lymphoid lineage cells (7, 8). Obesity has been associated with myeloid-biased hematopoiesis (aberrant myelopoiesis), which leads to an accumulation of circulating monocytes and contributes to chronic low-grade inflammation (9, 10). Preclinical work from our lab suggests that obesity-induced aberrant myelopoiesis is linked to higher CRC initiation potentially through the early accumulation of myeloid-derived pro-inflammatory cells in the colon (4, 11). Conversely, regular physical activity reduced chronic production of inflammatory monocytes in preclinical models of systemic inflammation (12). Further, exercise reversed the shift toward aberrant myelopoiesis in preclinical models of diet-induced obesity (13), likely by maintaining hematopoietic homeostasis and reducing the overproduction of inflammatory myeloid cells (14). Supporting translational relevance, in humans with obesity, Niemiro and colleagues determined that exercise training resulted in fewer inflammatory-primed HSPCs (15). In humans, aberrant myelopoiesis can be determined relatively non-invasively by the ratio of myeloid to lymphoid cell proportions from complete blood cell counts (16).

Large-scale epidemiological studies have established that higher adiposity and lower physical activity are associated with increased CRC risk in diverse populations (17–20). For example, using the Framingham Heart Study Dataset, Moore and colleagues demonstrated that BMI and waist circumference are independent predictors of lifetime colon cancer risk (21) while Ballard-Barbash and colleagues reported that lower physical activity increases large bowel cancer risk (20). Murphy and colleagues further showed that higher BMI is associated with increased risk of CRC across molecular subtypes (22), and recently, Safizadeh and colleagues reported that central obesity is a strong and significant predictor of CRC incidence (23). Meta-analyses have suggested that higher physical activity levels are linked to reduced CRC risk (19), and recently Laskar and colleagues reported adiposity and physical inactivity as associated risk factors of early onset CRC (24). Relatively little information exists that may mechanistically explain the divergent CRC risk between adults with obesity and those who regularly participate in physical activity. Meta-analyses confirm that chronic inflammation and immune cell profiles, including elevated monocyte counts, are linked to cancer incidence and prognosis in CRC and liver metastatic CRC (25, 26). In this context, the monocyte-to-lymphocyte ratio can be interpreted as a circulating inflammatory marker reflecting relative myeloid–lymphoid balance. Collectively, these findings support the hypothesis that myeloid skewing may link the opposing effects of adiposity and physical activity with CRC risk. However, this proposed inflammatory pathway has not been directly evaluated in humans, and it remains unknown the extent to which aberrant myelopoiesis may mediate increased CRC risk associated with higher BMI, waist circumference, and physical inactivity.

The present study used a secondary analysis of the Framingham Heart Study to explore the relationship between myeloid skewing, obesity, and physical activity levels in adults to determine the extent to which myeloid skewing is a predictor of CRC, while adjusting for clinical covariates and medication exposure. We hypothesized that myeloid skewing would increase with elevated BMI categories and waist circumference quartiles, decrease with increasing daily physical activity level, and be associated with CRC risk in multivariable models.

## 2 Materials and methods

### 2.1 Study population

The study population comprised the original, offspring, omni 1, gen 3, and omni 2 Framingham Heart Study cohorts. Each participant granted written informed consent as approved by the Institutional Review Board at Boston University Medical Center (H-32132). The present secondary analysis of de-identified Framingham Heart Study data was approved by the University of Ottawa Research Ethics Board (H-06-21-7100).

### 2.2 Variables

Participants underwent standardized questionnaires, physical examinations, anthropometric measurements, and blood sampling according to common Framingham protocols (17). At each visit, participants’ height and weight were measured and BMI was calculated as weight in kilograms divided by height in meters squared (17). Physical activity was assessed using the Framingham PAI, a validated questionnaire-based index that computes a weighted 24-hour score based on self-reported time spent in sleep, sedentary, light, moderate, and vigorous activities (18). Venous blood draws were collected as previously described for complete blood counts to derive myeloid skewing (17).

*BMI Categories:* BMI was categorized according to the World Health Organization (WHO) as underweight (<18.5 kg/m²), normal weight (18.5 - 24.9 kg/m²), overweight (25.0 - 29.9 kg/m²), and obese (≥30.0 kg/m²; 16, 20).

*Waist circumference quartiles:* Waist circumference measurements were stratified into quartiles (Q1 - Q4) based on the distribution across the entire study sample (21).

*PAI quintiles:* A weighted PAI was calculated using self-reported time spent in different activity categories. Activity hours were weighted according to metabolic intensity: sleep (1.0), sedentary activities (1.1), low-intensity activities (1.5), medium-intensity activities (2.4), and vigorous activities (5.0; 21). The total PAI score was computed as:


$$\begin{array}{c} PAI=\left(sleep\;hours\times 1.0\right)+\left(sedentary\;hours\times 1.1\right)\\ +\left(low-intensity\;hours\times 1.5\right)+\left(medium-intensity\;hours\times 2.4\right)\\ +\left(high-intensity\;hours\times 5.0\right) \end{array}$$

Within each sex, participants were classified into quintiles (Q1 - Q5) based on their PAI scores, where Q1 represents the lowest physical activity level and Q5 represents the highest (27).

*Myeloid skewing:* Myeloid skewing ratio was calculated as monocyte proportion divided by lymphocyte proportion from CBC counts. Higher values indicate greater myeloid skewing.

*Cancer diagnosis:* Colorectal cancer diagnosis was made as previously described (28), and histologically confirmed and coded according to the International Classification of Diseases for Oncology (ICD-O codes 153, 154; 29). Follow-up time (duration) was defined from baseline examination to CRC diagnosis or censoring and expressed in months.

*Additional covariates:* Additional covariates included age, sex, smoking status,diabetes status, and medications. Medication exposure was derived from ATC-coded records and grouped as diabetes medication, aspirin, systemic NSAIDs, and systemic glucocorticoids (**Supplementary Table 1**).

### 2.3 Statistical analysis

All statistical analyses were performed using Python (version 3.12.2), with statistical significance set at α = 0.05. One-way ANOVA was performed to compare markers of obesity (waist circumference quartile and BMI categories) across PAI quintile. Myeloid skewing (circulating myeloid cell: lymphoid cell ratio) was compared across BMI category, waist circumference quintile, and between the lowest PAI quintile (Q1) and two highest quintiles (Q4 + Q5; 22). Tukey’s Honestly Significant Difference (HSD) *post-hoc* tests were conducted to identify specific pairwise differences between quintiles/categories, with adjustment for multiple comparisons. Welch’s t-test was used to compare continuous variables between participants without and with a CRC diagnosis, and Fisher exact test was used to compare categorical variables between participants without and with a CRC diagnosis.

*Association of myeloid skewing with CRC risk:* A series of Cox proportional hazards models were fitted to evaluate the association between myeloid skewing and incident CRC. Myeloid skewing ratio was modeled as a continuous linear predictor, and primary effect estimates are reported per 1-SD increase. Time-to-event was defined from blood draw to CRC diagnosis or censoring (last follow-up), expressed in months. A series of Cox models were fitted, including unadjusted, and adjusted for age, sex, combined age and sex, BMI, waist circumference, combined BMI and waist circumference, PAI, diabetes status, smoking status, and reported medication exposure. Hazard ratios and 95% confidence intervals from the continuous Cox models were visualized using a forest plot. A Kaplan-Meier curve was used as a descriptive, unadjusted visualization based on myeloid-skewing quartiles (Q1-Q4), with a global log-rank test reported for the quartile comparison. Model-adjusted inference was based on the continuous Cox models rather than Kaplan-Meier stratification. Participants with missing values for any covariate included in multivariable models were excluded from those analyses. Cox model assumptions were assessed using scaled Schoenfeld residual tests and plots, martingale residuals with functional-form sensitivity analyses, influence diagnostics (deviance and delta-beta residuals), and collinearity diagnostics (VIF).

## 3 Results

### 3.1 Markers of myeloid skewing increased with obesity and decreased with physical activity

**Figure 1:**
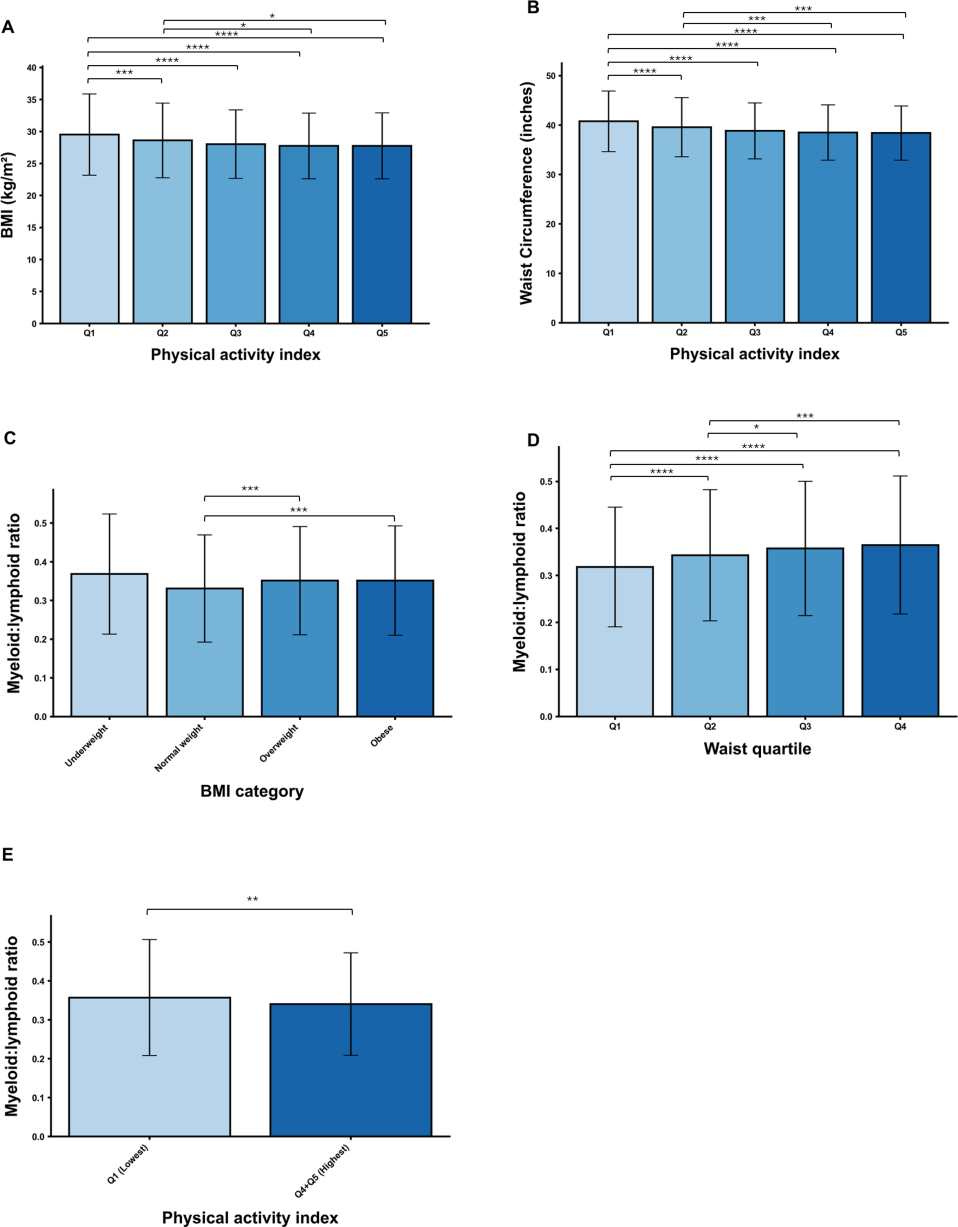
BMI and waist circumference decreased with increased physical activity score index sex-specific quintile, and myeloid skewing ratio increased with BMI, and waist circumference, and decreased with physical activity level. **(A)** BMI (kg/m^2^). **(B)** Waist circumference (inches). C, **(D)** Myeloid skewing ratio across BMI categories **(C)**, and across waist circumference quartiles **(D)**. Q1 = quartile 1, Q2 = quartile 2, Q3 = quartile 3, Q4 = quartile 4. **(E)** Myeloid skewing ratio between physical activity index quintile 1, and combined quintiles 4, and 5. Q1 = quintile 1, Q2 = quintile 2, Q3 = quintile 3, Q4 = quintile 4, Q5 = quintile 5. * P <.05, ** P <.01, *** P <.001, **** P <.0001.

Within the total study population (n = 5037; females 52.53%, mean age 57.85 ± 14.53 years (24 – 96; **Table 1**), BMI (Q5, Q4, and Q3 vs. Q1 P <.0001; Q2 vs Q1 P <.001; Q5 and Q4 vs. Q2 P = .0134, and.0104, respectively; **Figure 1A**) and waist circumference (Q5, Q4, Q3, and Q2 vs. Q1 P <.0001; Q5 and Q4 vs. Q2 P <.001; **Figure 1B**) decreased with sex-specific physical activity index quintile. Myeloid skewing increased with BMI (obese and overweight vs normal weight P <.001; **Figure 1C**), and with waist circumference (Q4, Q3, and Q2 vs. Q1 P <.0001; Q4 vs. Q2 P <.001; Q3 vs Q2 P <.05; **Figure 1D**). Conversely, myeloid skewing decreased with PAI between the lowest and highest quintiles (Q4 + Q5 vs. Q1; P < .01; **Figure 1E**).

**Table 1:** Baseline characteristics.

Variable	Group	N (male; female)	Age (years) Mean ± SD (Min–Max)	Myeloid skewing (ratio) Mean ± SD (Min–Max)
	Total	5037 (2391; 2646)	57.85 ± 14.53 (24–96)	0.35 ± 0.14 (0.05–1.53)
	Underweight	40 (3; 37)	59.98 ± 17.05 (29–90)	0.37 ± 0.16 (0.15–0.83)
BMI (kg/m²)	Normal weight	1443 (447; 996)	55.92 ± 15.47 (24–96)	0.33 ± 0.14 (0.06–1.53)
	Overweight	1936 (1084; 852)	59.03 ± 14.43 (25–92)	0.35 ± 0.14 (0.05–1.53)
	Obese	1618 (857; 761)	58.10 ± 13.51 (25–93)	0.35 ± 0.14 (0.05–1.28)
	Total	5037 (2391; 2646)	57.85 ± 14.53 (24–96)	
	Quartile 1	1277 (278; 999)	53.18 ± 14.75 (24–95)	0.32 ± 0.13 (0.06–1.38)
Waist circumference	Quartile 2	1263 (607; 656)	57.05 ± 14.74 (24–96)	0.34 ± 0.14 (0.07–1.53)
(inches)	Quartile 3	1245 (758; 487)	60.69 ± 13.96 (25–92)	0.36 ± 0.14 (0.05–1.53)
	Quartile 4	1252 (748; 504)	60.58 ± 13.29 (26–93)	0.36 ± 0.15 (0.05–1.28)
	Total	5037 (2391; 2646)	57.85 ± 14.53 (24–96)	
	Quintile 1	1021 (483; 538)	61.57 ± 15.50 (24–96)	0.356 ± 0.149 (0.051–1.532)
Physical Activity Index	Quintile 2	1022 (477; 545)	58.07 ± 15.20 (26–92)	0.346 ± 0.141 (0.106–1.529)
	Quintile 3	988 (477; 511)	57.47 ± 14.05 (24–91)	0.345 ± 0.148 (0.061–1.379)
	Quintile 4	1015 (478; 537)	56.67 ± 13.65 (24–91)	0.345 ± 0.136 (0.055–1.439)
	Quintile 5	991 (476; 515)	55.35 ± 13.35 (25–87)	0.337 ± 0.127 (0.071–1.149)

Age and sex by BMI (kg/m^2^), waist circumference (inches), and physical activity index, and circulating myeloid skewing ratio by BMI category, waist circumference quartile, and physical activity index quintile.

BMI, body mass index; SD, standard deviation.

### 3.2 Higher myeloid skewing is associated with increased CRC risk in continuous Cox models

In descriptive baseline analyses, baseline myeloid skewing was higher among participants already diagnosed with CRC before baseline (0.39 ± 0.15, n = 42; duration < 0) than among non-CRC participants (0.34 ± 0.14, n = 2228; P = .0265; **Table 2**). In the predictive cohort (duration > 0), baseline myeloid skewing was numerically higher in participants who were subsequently diagnosed with CRC (0.37 ± 0.18, n = 16) than in non-CRC participants (0.31 ± 0.12, n = 818), but this difference was not statistically significant (P = .2102; **Table 2**).

**Table 2:** Baseline characteristics for participants with and without CRC.

Variable	Level	Non-CRC	CRC	Total	P value
Continuous variable		Mean ± SD (Min–Max)	Mean ± SD (Min–Max)		
Myeloid skewing ratio (diagnosed)		0.34 ± 0.14 (0.03-1.44) N = 2228	0.39 ± 0.15 (0.17-0.73) N = 42		0.0265*
Duration (months) (diagnosis to baseline)		-68.26 ± 242.62 (-823.59- -467.21) N = 2228	-177.93 ± 128.81 (-456.77 - -9.53) N = 42		.0001***
Myeloid skewing ratio (predictive)		0.31 ± 0.12 (0.03-1.44) N = 818	0.37 ± 0.18 (0.07-0.68) N = 16		0.2102
Duration (months) (baseline to diagnosis)		174.72 ± 130.86 (0.03 - 467.21) N = 818	195.08 ± 136.09 (22.50 - 455.91) N = 16		0.5618
Age		56.33 ± 14.33 (24.00-88.00) N = 818	59.69 ± 14.89 (39.00-87.00) N = 16		0.3848
BMI		20.99 ± 12.97 (0.00-50.78) N = 818	28.03 ± 10.16 (0.00-45.44) N = 16		0.0150*
Waist		28.79 ± 17.31 (0.00-59.50) N = 818	37.50 ± 11.94 (0.00-54.00) N = 16		0.0112*
PAI		14.04 ± 6.10 (1.00-36.00) N = 818	12.81 ± 7.07 (3.00-27.00) N = 16		0.5004
Meds total		0.24 ± 0.46 (0.00-3.00) N = 818	0.31 ± 0.48 (0.00-1.00) N = 16		0.5417
Duration		5318.62 ± 3983.22 (1.00-14222.00) N = 818	5938.12 ± 4142.55 (685.00-13878.00) N = 16		0.5618
Categorical Variable		N	N		
Sex	Male	349/818 (42.7%)	5/16 (31.2%)	354/834 (42.4%)	0.4488
	Female	469/818 (57.3%)	11/16 (68.8%)	480/834 (57.6%)	
Smoking	Smoking	42/818 (5.1%)	1/16 (6.2%)	43/834 (5.2%)	0.5747
	Non-smoking	776/818 (94.9%)	15/16 (93.8%)	791/834 (94.8%)	
Diabetes	Diabetes	104/818 (12.7%)	4/16 (25.0%)	108/834 (12.9%)	0.1414
	No diabetes	714/818 (87.3%)	12/16 (75.0%)	726/834 (87.1%)	
Meds	≥1 med group	184/818 (22.5%)	5/16 (31.2%)	189/834 (22.7%)	0.3773
	0 med groups	634/818 (77.5%)	11/16 (68.8%)	645/834 (77.3%)	
Diabetes medication	Yes	18/818 (2.2%)	2/16 (12.5%)	20/834 (2.4%)	0.0536
	No	800/818 (97.8%)	14/16 (87.5%)	814/834 (97.6%)	
Aspirin	Yes	21/818 (2.6%)	0/16 (0.0%)	21/834 (2.5%)	1.0000
	No	797/818 (97.4%)	16/16 (100.0%)	813/834 (97.5%)	
Systemic NSAID	Yes	149/818 (18.2%)	3/16 (18.8%)	152/834 (18.2%)	1.0000
	No	669/818 (81.8%)	13/16 (81.2%)	682/834 (81.8%)	
Systemic glucocorticoid	Yes	6/818 (0.7%)	0/16 (0.0%)	6/834 (0.7%)	1.0000
	No	812/818 (99.3%)	16/16 (100.0%)	828/834 (99.3%)	

*P <.05, ***P <.0001. CRC, colorectal cancer; BMI, body mass index; PAI, physical activity index; NSAID, nonsteroidal anti-inflammatory drug; SD, standard deviation.

Continuous variables; P values are from Welch t-tests. Categorical variables: P values are from Fisher exact tests (two-sided). Meds total any (≥1 med group) indicates presence of at least one medication group.

**Figure 2:**
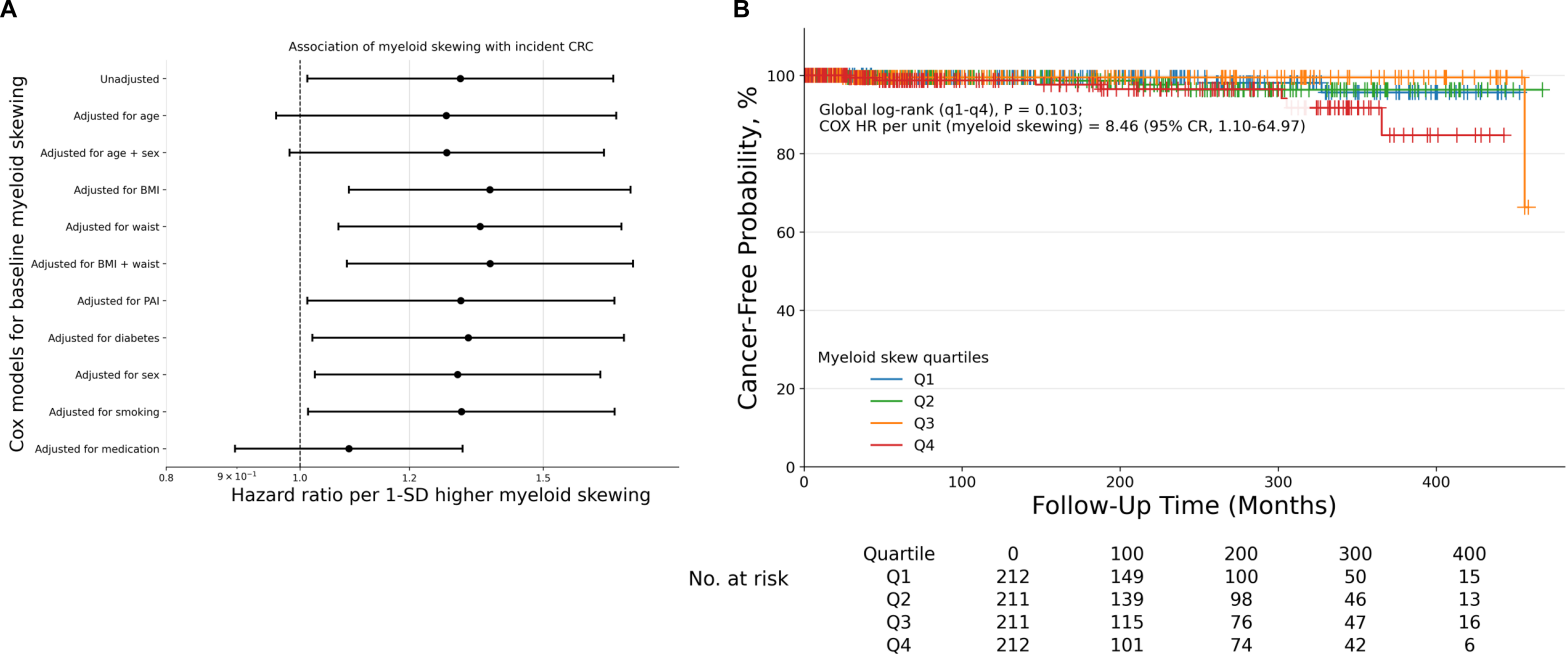
Myeloid skewing and incident CRC risk in continuous Cox and Kaplan-Meier analyses. **(A)** Forest plot summarizing hazard ratios and 95% confidence intervals for incident CRC per 1-SD increase in baseline myeloid skewing across unadjusted and adjusted continuous Cox proportional hazards models. Each estimate represents the association between myeloid skewing and incident CRC, with the y-axis indicating the covariates included in each model. **(B)** Unadjusted Kaplan-Meier curve showing cancer-free probability by baseline myeloid-skewing quartiles (Q1-Q4), with global log-rank P value reported for the quartile comparison. BMI = Body Mass Index (kg/m^2^), CRC = colorectal cancer, PAI = Physical Activity Index, Waist = Waist circumference (inches).

Continuous Cox models showed positive associations between higher myeloid skewing and CRC risk in an unadjusted model (1.3062 CI 1.0123-1.6856, P = .0400), and several adjusted models. including BMI-adjusted (HR 1.3722, 95% CI 1.0851-1.7352, P = .0082), waist circumference-adjusted (HR 1.3498, 95% CI 1.0662-1.7090, P = .0127), combined BMI and waist (HR 1.3727, 95% CI 1.0813-1.7426, P = .0093), PAI-adjusted (HR 1.3074, 95% CI 1.0121-1.6889, P = .0402), diabetes-adjusted (HR 1.3235, 95% CI 1.0206-1.7163, P = .0345), sex-adjusted (HR 1.3002, 95% CI 1.0248-1.6497, P = .0307), and smoking-adjusted (HR 1.3087, 95% CI 1.0135-1.6899, P = .0391; **Table 3**). Associations were directionally consistent but not statistically significant in age-adjusted, and combined age and sex-adjusted models, and were attenuated in the medication-adjusted model (HR 1.0847, 95% CI 0.8973-1.3112, P = .4007; **Table 3**). Model-specific hazard ratios and 95% confidence intervals are additionally summarized in a forest plot (**Figure 2A**). In descriptive unadjusted Kaplan-Meier analysis by myeloid quartiles (Q1-Q4), curve separation was not statistically significant (global log-rank P = .103; **Figure 2B**). Assumption checks showed no evidence of major proportional hazards or multicollinearity violations across models (all VIFs ≤ 1.10). Influence diagnostics identified some influential observations, and estimates were interpreted cautiously given sparse events.

**Table 3:** Cox-proportional hazards model reporting myeloid skewing ratio and risk of colorectal cancer.

Model	Covariate	HR [95% CI]	P value	Concordance	Partial AIC
Unadjusted	myeloid skew	1.3062 [1.0123-1.6856]	.0400	0.612	177.36
Age (years)	myeloid skew	1.2761 [0.9611-1.6944]	.0919	0.724	176.86
	age	1.0293 [0.9927-1.0673]	.1184	0.724	176.86
Age + Sex	myeloid skew	1.2771 [0.9828-1.6596]	.0673	0.7505	177.8163
	age	1.0283 [0.9916-1.0664]	.1323	0.7505	177.8163
	sex	1.7144 [0.5921-4.9640]	.3204	0.7505	177.8163
BMI (kg/m²)	myeloid skew	1.3722 [1.0851-1.7352]	.0082**	0.6626	156.9429
	BMI	1.0696 [0.9854-1.1609]	.1077	0.6626	156.9429
Diabetes	myeloid skew	1.3235 [1.0206-1.7163]	.0345*	0.6137	176.9678
	diabetes	2.6532 [0.8391-8.3899]	.0967	0.6137	176.9678
Medication	myeloid skew	1.0847 [0.8973-1.3112]	.4007	0.6462	185.7443
	any diabetes med	2.1480 [0.6126-7.5313]	.2323	0.6462	185.7443
	any aspirin	0.7351 [0.2194-2.4632]	.6179	0.6462	185.7443
	systemic NSAID	1.0196 [0.6120-1.6985]	.9407	0.6462	185.7443
	systemic glucocorticoid	0.7837 [0.0803-7.6513]	.8339	0.6462	185.7443
PAI	myeloid skew	1.3074 [1.0121-1.6889]	.0402*	0.6472	179.2436
	PAI	0.9853 [0.9035-1.0745]	.7375	0.6472	179.2436
Sex	myeloid skew	1.3002 [1.0248-1.6497]	.0307*	0.6172	178.1336
	sex	1.7906 [0.6193-5.1776]	.2822	0.6172	178.1336
Smoking	myeloid skew	1.3087 [1.0135-1.6899]	.0391*	0.601	179.1526
	Smoking	1.5357 [0.2014-11.7120]	.679	0.601	179.1526
Waist Circumference (inches)	myeloid skew	1.3498 [1.0662-1.7090]	.0127*	0.6715	157.3194
	Waist	1.0624 [0.9776-1.1545]	.1538	0.6715	157.3194
BMI + Waist	myeloid skew	1.3727 [1.0813-1.7426]	.0093**	0.6624	158.9182
	BMI	1.0721 [0.8649-1.3290]	.5252	0.6624	158.9182
	Waist	0.9977 [0.8057-1.2356]	.9834	0.6624	158.9182

*P <.05, **P <.01.

AIC, akaike information criterion; HR, hazard ratio; CI, confidence interval.

## 4 Discussion

Aberrant myelopoiesis has been proposed as a biologically plausible pathway linking obesity, physical activity, and CRC risk in preclinical models (4, 11). Large epidemiological studies have likewise shown that higher adiposity and lower physical activity are associated with increased CRC risk in humans (17, 19, 20, 30). However, no previous studies have determined if myeloid skewing represents a measurable link between adiposity, physical inactivity, and CRC risk in humans. In this exploratory analysis of the Framingham Heart Study, we examined circulating myeloid cell skewing in relation to adiposity, physical activity, and incident CRC risk. As expected, we found elevated myeloid skewing across BMI categories and waist circumference quartiles, and lower myeloid skewing with higher PAI quintiles. Interestingly, higher baseline myeloid skewing was associated with increased incident CRC risk in several adjusted Cox models. Together, these findings extend preclinical and epidemiological literature by supporting myeloid skewing as a candidate blood-based marker associated with lifestyle-related CRC risk.

Obesity has been associated with myeloid skewing in human studies and animal models (4, 11, 31). We extend these findings in a large cohort by showing that the obesity markers, BMI and waist circumference, were associated with higher myeloid skewing. We also observed that lower physical activity was associated with higher myeloid skewing, as participants in lower sex-specific PAI quintiles had greater myeloid skewing than those in higher quintiles. However, the small absolute differences suggest that clinical relevance of these findings may be limited despite statistical significance. Regardless, this pattern remains consistent with prior literature suggesting that high physical activity and exercise training can reduce obesity-associated myeloid skewing by limiting the expansion of common myeloid progenitor cells (11, 15). Prior human studies also suggest that modest variation in CBC-derived myeloid-lymphoid balance may still have prognostic relevance. In CRC, low lymphocyte-to-monocyte ratio has been associated with worse survival in meta-analyses, while neutrophil-to-lymphocyte ratio is reported to predict mortality independent of conventional risk factors (32, 33). Thus, despite small differences that reach statistical significance, our findings align with previous work showing the clinical relevance of these findings.

Myeloid cell accumulation in the colon has been associated with CRC in preclinical models, with aberrant myelopoiesis suggested as a mechanism responsible for early CRC initiation in obesity (11). Thus, we examined the extent to which baseline myeloid skewing was associated with incident CRC risk in humans. Interestingly, we found that myeloid skewing was associated with increased CRC risk in several adjusted continuous models, supporting the biological plausibility of myeloid skewing as a blood-based marker of CRC risk. Specifically, significant associations were observed in BMI, waist circumference, combined BMI and waist circumference, PAI, diabetes status, sex, and smoking status adjusted models (HRs 1.30 to 1.37). These estimates were modeled per 1-SD increase in baseline myeloid skewing (HR 1.30 to 1.37); therefore, these findings suggest that each 1-SD increase in baseline myeloid skewing is associated with a 30% to 37% higher risk of CRC across significant adjusted models. This is consistent with previous prospective work showing that elevations in CBC-derived inflammatory indices are associated with incident CRC risk in large cohorts including a negative association with lymphocyte-to-monocyte ratio (0.70-1.00; 34). By comparison, elevated BMI is associated with an increase in CRC risk of 1.18 times (1.15 to 1.22; 26). In the Framingham cohort, BMI ≥30 kg/m², and waist circumference >39 inches in males and >40 inches in females increased CRC risk by 2.5 times (1.4 to 3.9), and 2.6 times (1.3 to 5.2), respectively (21). Higher physical activity is inversely associated with colon cancer risk by 0.76 times (0.72 to 0.81; 35), whereas low physical activity in the Framingham cohort increased CRC risk in males by 1.8 times (1.0 to 3.2; 17). Among cigarette smokers, meta-analyses have reported relative risks of CRC incidence from 1.18 to 1.20 times (36). Our findings therefore extend existing epidemiological literature by suggesting that myeloid skewing may also be associated with CRC risk prior to diagnosis, and that myeloid skewing is a relevant indicator of CRC risk.

Not all adjusted models showed statistically significant associations between myeloid skewing and incident CRC risk. Associations were attenuated in the age-adjusted, combined age and sex-adjusted, and medication-adjusted models, although effect estimates remained directionally consistent with the significant models (HRs 1.08 to 1.28). This pattern suggests that age, sex, and medication exposure may partly confound or overlap with the observed association between myeloid skewing and CRC risk. It also indicates that the relationship was sensitive to demographic and clinical adjustment, because sparce events led to an uncertain estimate that changed after model adjustment. Together, these results support cautious interpretation and indicate that myeloid skewing may represent an exploratory marker of CRC risk.

Biologically, myeloid skewing may reflect a relative increase in monocyte-derived myeloid cells, which has been implicated in tissue infiltration and macrophage accumulation during early CRC stages (11, 37, 38). Macrophage infiltration in the colon is associated with greater inflammation, carcinogenesis, CRC initiation, and a worse CRC prognosis (39–41). A meta-analysis of 16 included studies from Wen and colleagues examined patients with established CRC and revealed that an elevated absolute monocyte count was significantly associated with worse overall survival, disease free survival, and cancer specific survival (25), while Yang and colleagues reported elevated absolute monocyte count as significantly associated with an increased risk of colorectal liver metastasis (26). Our findings complement this existing literature by suggesting that higher circulating myeloid skewing may also be associated with CRC risk prior to diagnosis, further supporting a possible role for myeloid lineage imbalance across CRC incidence, initiation, prognosis, and outcome.

### 4.1 Limitations

The primary limitation of our study is the small number of CRC cases, which limit statistical power and contribute to imprecision in hazard ratio estimates. We acknowledge that in sparse-event Cox regression settings, effect estimates may be unstable and sensitive to model specification, particularly when multiple adjustment sets are examined (42–45). Accordingly, the possibility of type I error cannot be excluded, and the observed associations should be interpreted as exploratory rather than definitive. Although the Framingham Heart Study includes thousands of participants, circulating blood data required to calculate myeloid skewing is only available in more recent cohorts, restricting the analytic sample. As a result, sample sizes were limited to n = 42 for participants diagnosed with CRC before baseline blood draw and n = 16 for those diagnosed after baseline blood draw. Residual confounding also remains possible despite adjustment for age, sex, adiposity, physical activity, smoking, diabetes, and medication exposure, as other relevant factors such as diet, family history of CRC, baseline inflammatory conditions, and more detailed medication characterization were not fully captured. Finally, the relative homogeneity of the cohort may limit generalizability to more diverse populations. Despite these limitations, our findings remain consistent with the literature reporting elevated myeloid lineage cells as promising prognostic tools for CRC diagnosis and progression (25, 26, 46). As the Framingham dataset grows and more participants from this subpopulation become available, larger prospective studies with more CRC events and broader confounder assessment are warranted to validate these findings.

### 4.2 Conclusions

Taken together, our results suggest that lifestyle factors, such as greater adiposity and lower physical activity, are associated with higher myeloid skewing, and that higher myeloid skewing is associated with increased incident CRC risk. Additionally, we found that higher adiposity and lower physical activity were associated with myeloid skewing, which is consistent with previous Framingham Heart Study analyses identifying obesity and physical inactivity as CRC risk predictors (20, 21). Crucially, given the increasing prevalence of CRC in young adults who are below the age of regular preventative screening (2), our exploratory findings support myeloid skewing as a candidate immune-related marker of CRC risk.

## Data Availability

The datasets presented in this study can be found in online repositories. The data analyzed in this study were obtained from the Framingham Heart Study via the NHLBI controlled-access repository (BioLINCC). Restrictions apply under the Framingham Heart Study data use agreements. Requests to access these datasets should be directed to NHLBI BioLINCC.

## Supplementary material

Supplementary Table 1 Medication group definitions and ATC-coded medication use for participants with and without CRC. Medication groups used in Cox models: diabetes medications, aspirin, systemic NSAID, systemic glucocorticoid. ATC names are based on standard WHO ATC descriptions; class-level codes are labeled as class.
