# Supplementary material for: Relationship between myeloid skewing and colorectal cancer risk in the Framingham Heart Study
Source: Front Oncol. 2026 Apr 22;16:1773537. doi: 10.3389/fonc.2026.1773537 (PMC13143660; doi:10.3389/fonc.2026.1773537)
Supplement: Supplementary Table 1 — Medication group definitions and ATC-coded medication use for participants with and without CRC. Medication groups used in Cox models: diabetes medications, aspirin, systemic NSAID, systemic glucocorticoid. ATC names are based on standard WHO ATC descriptions; class-level codes are labeled as class. [file Table1.docx]

**Supplemental Table S1.** Medication group definitions and ATC-coded medication use for participants with and without CRC.

| **Medication Group** | **ATC Code** | **Medication/Class Name** | **Non-CRC (N)** | **CRC (N)** | **Total (N)** |
| --- | --- | --- | --- | --- | --- |
| Aspirin | B01AC06 | Aspirin (antiplatelet) | 155 | 0 | 155 |
| Aspirin | N02BA01 | Aspirin (analgesic/antipyretic) | 3 | 0 | 3 |
| Diabetes medication | A10A | Insulins and analogues (class) | 13 | 0 | 13 |
| Diabetes medication | A10AB01 | Insulin (human), fast-acting | 8 | 0 | 8 |
| Diabetes medication | A10AB04 | Insulin lispro | 31 | 3 | 34 |
| Diabetes medication | A10AB05 | Insulin aspart | 2 | 0 | 2 |
| Diabetes medication | A10AB06 | Insulin glulisine | 2 | 0 | 2 |
| Diabetes medication | A10AC01 | Insulin (human), intermediate-acting | 4 | 0 | 4 |
| Diabetes medication | A10AD02 | Insulin combinations (class) | 1 | 0 | 1 |
| Diabetes medication | A10AD04 | Insulin lispro + lispro protamine | 1 | 0 | 1 |
| Diabetes medication | A10AD05 | Insulin aspart + aspart protamine | 14 | 1 | 15 |
| Diabetes medication | A10AE | Long-acting insulins and analogues (class) | 2 | 0 | 2 |
| Diabetes medication | A10AE01 | Insulin (human), long-acting | 8 | 0 | 8 |
| Diabetes medication | A10AE04 | Insulin glargine | 57 | 3 | 60 |
| Diabetes medication | A10AE05 | Insulin detemir | 7 | 1 | 8 |
| Systemic NSAID | M01AA01 | Phenylbutazone | 1 | 0 | 1 |
| Systemic NSAID | M01AB01 | Indometacin | 20 | 2 | 22 |
| Systemic NSAID | M01AB02 | Sulindac | 8 | 0 | 8 |
| Systemic NSAID | M01AB05 | Diclofenac | 35 | 1 | 36 |
| Systemic NSAID | M01AB08 | Etodolac | 7 | 0 | 7 |
| Systemic NSAID | M01AB15 | Ketorolac | 1 | 0 | 1 |
| Systemic NSAID | M01AB55 | Diclofenac combinations | 1 | 0 | 1 |
| Systemic NSAID | M01AC01 | Piroxicam | 9 | 0 | 9 |
| Systemic NSAID | M01AC06 | Meloxicam | 23 | 1 | 24 |
| Systemic NSAID | M01AE | Propionic acid derivatives (class) | 1 | 0 | 1 |
| Systemic NSAID | M01AE01 | Ibuprofen | 735 | 4 | 739 |
| Systemic NSAID | M01AE02 | Naproxen | 255 | 2 | 257 |
| Systemic NSAID | M01AE09 | Flurbiprofen | 1 | 0 | 1 |
| Systemic NSAID | M01AE11 | Tiaprofenic acid | 1 | 0 | 1 |
| Systemic NSAID | M01AE12 | Oxaprozin | 1 | 0 | 1 |
| Systemic NSAID | M01AG01 | Mefenamic acid | 1 | 0 | 1 |
| Systemic NSAID | M01AH01 | Celecoxib | 51 | 0 | 51 |
| Systemic NSAID | M01AX01 | Nabumetone | 9 | 0 | 9 |
| Systemic NSAID | M01AX05 | Glucosamine | 399 | 4 | 403 |
| Systemic NSAID | M01AX25 | Other anti-inflammatory/antirheumatic agent | 50 | 0 | 50 |
| Systemic glucocorticoid | H02AB02 | Dexamethasone | 3 | 0 | 3 |
| Systemic glucocorticoid | H02AB04 | Methylprednisolone | 3 | 0 | 3 |
| Systemic glucocorticoid | H02AB06 | Prednisolone | 2 | 0 | 2 |
| Systemic glucocorticoid | H02AB07 | Prednisone | 58 | 2 | 60 |
| Systemic glucocorticoid | H02AB08 | Triamcinolone | 2 | 0 | 2 |
| Systemic glucocorticoid | H02AB09 | Hydrocortisone | 3 | 0 | 3 |
| Systemic glucocorticoid | H02AB10 | Cortisone | 3 | 0 | 3 |

Note: Medication groups used in Cox models: diabetes medications, aspirin, systemic NSAID, systemic glucocorticoid. ATC names are based on standard WHO ATC descriptions; class-level codes are labeled as class.
